# Spatial and Compositional Variations in Fruit Characteristics of Papaya (*Carica papaya* cv. Tainung No. 2) during Ripening

**DOI:** 10.3390/plants12071465

**Published:** 2023-03-27

**Authors:** Sun Woo Chung, Yeon Jin Jang, Seolah Kim, Seong Cheol Kim

**Affiliations:** Research Institute of Climate Change and Agriculture, National Institute of Horticultural and Herbal Science, Jeju 63240, Republic of Korea; jsw599@korea.kr (S.W.C.);

**Keywords:** *Carica papaya*, cultivation practice, fruit development, fruit tree, greenhouse, mineral distribution, papaya fruit, skin coloration, nutrient, ripening stages, tropical

## Abstract

Papaya fruit (*Carica papaya*) has different degrees of ripening within each fruit, affecting its commercial market value. The fruit characteristics of “Tainung No. 2” Red papaya were investigated at the stem-end, middle, and calyx-end across 3 ripening stages and categorized based on fruit skin coloration: unripe at 16 weeks after anthesis (WAA), half-ripe at 18 WAA, and full-ripe at 20 WAA. The fruits maintained an elliptical shape during ripening with a ratio of 2.36 of the length to the width. The peel and pulp color changed from green to white to yellow during ripening, regardless of the three parts. In the pulp, soluble solid contents increased, and firmness decreased during ripening but did not differ among the three parts. Individual nutrient contents, including metabolites and minerals, changed dynamically between the ripening stages and fruit parts. Total carbohydrates and proteins, N, and K, were accumulated more at the stem-end during ripening; meanwhile, fructose, glucose, Mg, and Mn were accumulated more at the calyx-end. In the principal component analysis, ripening stages and fruit parts were distinctly determined by the first and second principal components, respectively. Understanding the nutrient and metabolite dynamics during ripening and their distribution within the fruit can help optimize cultivation practices, enhance fruit quality, and ultimately benefit both growers and consumers.

## 1. Introduction

Papaya (*Carica papaya*) is a popular fruit crop known for its sweet and distinct flavor. The global production of papaya has continued to grow over the years, with an estimated production of 14.10 million metric tons in 2021, making it the fourth most widely cultivated tropical fruit behind banana, mango, and pineapple [1]. Papaya originated from the lowland of Mexico to Panama and is found primarily in tropical regions between 23° N and S latitudes because the cultivation requires high temperatures year-round [2]. Subtropical regions, including Mediterranean countries, can also cultivate papaya; some of these regions have to use protected facilities, including a greenhouse, because low temperatures during winter affect fruit set, growth, and production [3,4,5]. Recently, due to the increasing demand for papaya and the changing climate, the cultivation has expanded to temperate regions such as Far East Asia [6,7,8].

Papaya fruits have nutraceutical values, which render them important in human diets. These papaya parts are rich in macro- and micronutrients to varying degrees [9]. Papaya fruit is abundant in bioactive compounds, including phenolics, carotenoids, saponins/triterpenoids, and ascorbic acid, which possess various medicinal properties [2]. Papaya fruits exhibit cell protection against oxidative stress, which causes or progresses several diseases, such as cancer, metabolic disorders, and cardiovascular diseases [9]. In addition, papaya fruit displays anti-inflammatory properties by regulating signaling pathways such as NF-κB and MAPKs. NF-κB, a transcription factor, plays a role in the production of various pro-inflammatory mediators such as iNOS, COX-2, and cytokines [7]. The buildup of excessive NO and increased COX-2 expression are linked to human diseases such as cancer and inflammation. Research on the bioactive components of papaya fruit is needed to fully understand the extent of their benefits for human health. 

Papaya fruit quality is determined by various characteristics that are required from the market. Papaya fruits have different shapes as sex forms, including males, females, and hermaphrodites. Markets prefer the hermaphrodite fruit shape, which tends to be pear-shaped or elongated [2]. The nutritional value of papaya fruit depends on the fruit ripening stages. Papaya fruit ripening is accompanied by physiochemical changes, including sugar metabolism, peel color changes, and pulp softening [10]. The more ripened fruit shows different physicochemical characteristics, such as moisture, titratable acidity, and soluble solid contents, as well as higher antioxidant activity and higher total phenolic and flavonoid contents [7,11]. Minerals, according to the fruit ripening progress, have different distributions depending on the fruit ripening stage [12]. The fruit is consumed regardless of ripeness and can be eaten either raw or in processed forms [9], with unripe fruits being used as a vegetable and ripe fruits being used as fruit [4].

Ripening initiation is a critical stage that varies significantly across different fruit species, resulting in uneven ripening [13,14]. It has been reported that the initiation of ripening occurs in specific fruit regions, causing distinct color changes [14,15]. For instance, tomatoes start ripening near the calyx [14], bananas start from their distal ends [15], and apples begin ripening from the stem-end [13]. Variations in fruit qualities have also been observed in apples [13,16], bananas [15], and grapes [17]. These differences could be attributed to genotypes and environmental factors, such as sun exposure and fruit skin temperatures [13]. The different ripening degrees of fruit parts may lead to a loss of consumer confidence [13,18,19]. Uneven fruit skin color has been reported in papaya [18], but the other characteristics have yet to be well studied in different parts.

In this study, we investigated morphological, physicochemical, and nutritional characteristics in different parts, ranging from the stem-end to the calyx-end, of “Tainung No.2” papaya fruits during ripening. We also investigated the relationship between the fruit characteristics, parts, and ripening stages. The fruit characteristics and their relationships would be contributed to a commercially valuable database associated with papaya fruit physiology.

## 2. Results

The fruit length, the width, the ratio of the length to the width, and the weight did not change during ripening (Table 1). The length of the papaya fruit was an average of 23.6 mm, ranging from 19.2 to 25.8 mm; the width was, on average, 10.0 mm, ranging from 9.6 to 10.3 mm. The ratio of the length to width ranged from 1.9 to 2.7, indicating the elliptical shape of the papaya fruit. The fresh weight of the fruit ranged from 699.6 to 997.9 g, with an average of 854.5 g.

**Figure 1:**
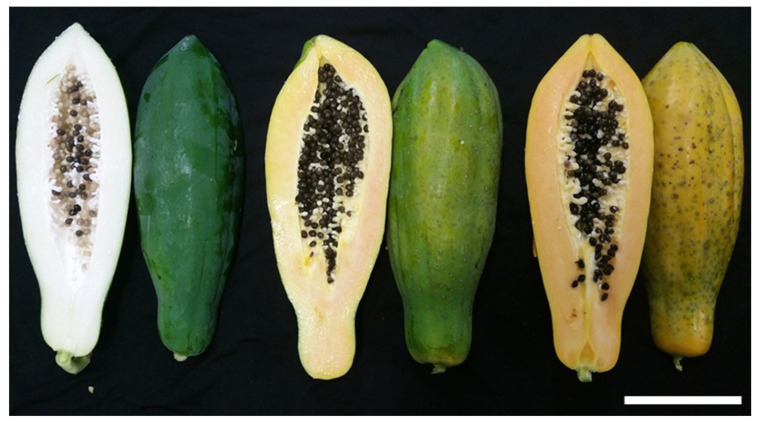
Inner and outer flesh of papaya (*Carica papaya*) fruit across different ripening stages: unripe (**left**), half-ripe (**middle**), full-ripe (**right**). Bar = 10 mm.

The peel color of the papaya fruit changed during ripening (Figure 1 and Table 2). The peel lightness decreased at the half-ripe and full-ripe stages compared with the unripe stage (Table 2). The half-ripe and full-ripe stages became redder during ripening than in the unripe stage. The b value increased during ripening, indicating that the pulp became yellow. The h° between the parts did not change at each ripening stage. The h° decreased in the half-ripe and full-ripe stages compared to that in the unripe stage; the values were, on average, 129.9, 75.5, and 58.0 at the unripe, half-ripe, and full-ripe stages, respectively. The h° of the unripe stage was significantly higher than those of the other two stages. The h° tended to decrease during fruit ripening continuously. The trend was obviously shown in the calyx-end parts of each stage, in which the h° significantly decreased. The C* increased in the half-ripe and full-ripe stages (51.9 and 60.0, respectively) compared to the unripe stage (13.2); there was no significant difference between the half-ripe and full-ripe stages.

Pulp characteristics were investigated during fruit parts and ripening (Table 3). The pulp color changed from unripe to half-ripe stages (Table 3) but not afterward. The lightness became darker in the half-ripe and full-ripe stages (55.8) than in the unripe stages (75.1). The a* and b* values of the unripe stage were lower than those of the half-ripe and full-ripe stages, indicating a greenish and a yellowish color, respectively. The significance of the h° and C* during ripening was consistent with the L, a*, and b* values. Soluble solid contents did not change between the fruit parts at each ripening stage but changed significantly during ripening, from the unripe stage (3.9 °Brix) to the half-ripe and full-ripe stages (10.8 °Brix). Pulp firmness did not change at the fruit parts in each ripening stage. The firmness did not change between the unripe and half-ripe stages and decreased only at the full-ripe stage up to 96% (from 9.75 N to 0.37 N).

The water contents continuously decreased during fruit ripening (Table 4). The water contents of the unripe, half-ripe, and full-ripe stages were 93.9, 92.5, and 91.2%, respectively. The water content was not different among the parts of the unripe and half-ripe stages. In the full-ripe stage, the content was the lowest in the stem-end (90.6%) over the other parts (91.5%). The total carbohydrates tended to increase during fruit ripening, showing the differences among the parts (Table 4). The lowest amount was 4.5 g/100 g at the stem-end and the calyx-end of the unripe stage; conversely, the highest was 7.4 g/100 g at the stem-end of the full-ripe stage. The accumulation rate of carbohydrates at the stem-end increased from 120% to 137% during ripening, while the rate at the middle and calyx-end ranged from 120% to 110% and from 131% to 117%, respectively. Protein contents did not show distinct tendencies during ripening in part of the fruit, except that the accumulation from the stem-end of each stage was higher than that in the other parts. In addition, the lipid contents were not detected in any fruit part during the ripening. 

Three soluble sugars were determined, and two were detected during ripening (Table 5). Sugar contents increased during ripening and were different from that of the fruits. Fructose contents were not different among the parts at the unripe stage: the fructose contents in the calyx-end of the two other stages were approximately 110% higher than that in the middle and stem-end. The glucose content in each part of the fruit significantly increased during ripening. However, these contents were not different at the three parts within each ripening stage, except for the full-ripe stage, in which the glucose content of the calyx-end was higher than that of the stem-end.

In addition to these structural compounds, bioactive compounds and minerals are essential components of fruits. In the papaya fruit, ascorbic acid contents increased up to approximately 300% from unripe to half-ripe and approximately 140% from half-ripe to full-ripe in all 3 parts during ripening (Table 6). However, there were no differences between the ripening stages. The proportion of mineral compounds varied between fruit parts and ripening stages (Table 7 and Table 8). The K proportion was about 2.85% during ripening, followed by N (0.80%), P (0.37%), Ca (0.24%), Mg (0.13%), Fe (26.05 ppm), Cu (2.73 ppm), Zn (11.11 ppm), and Mn (1.13 ppm). The proportion of K in each part was maintained during ripening, whereas those of other minerals fluctuated during ripening. The change patterns of the N proportion were distinctly different for each part: increase at the stem-end in the full-ripe stage, decrease at the middle in the half-ripe stage, decrease and then increase at the calyx-end in the half-ripe and full-ripe stage, respectively. The P proportion changed only at the stem-end between the unripe and full-ripe stages. The Ca proportion steadily decreased at the stem-end and middle parts during ripening; that of the calyx-end decreased in the half-ripe stage and was maintained up to the full-ripe stage. The Mg proportion of all fruit parts decreased from the unripe to half-ripe stages and then remained in the full-ripe stage. The Fe proportion of all the parts decreased from the unripe to half-ripe stages. Subsequently, the Fe proportion of the stem-end did not change, and those of the middle and calyx-end increased at the full-ripe stage. The Cu proportion decreased only in the calyx-end from the unripe to half-ripe stages; all the other proportions did not differ between the parts and ripening stages. The Zn proportion decreased from the unripe to the half-ripe stage and was maintained between the parts and ripening stages. The Mn proportion at the stem-end did not change during ripening, that at the middle increased only in the full-ripe stage, and that at the calyx-end decreased and then increased during ripening.

**Figure 2:**
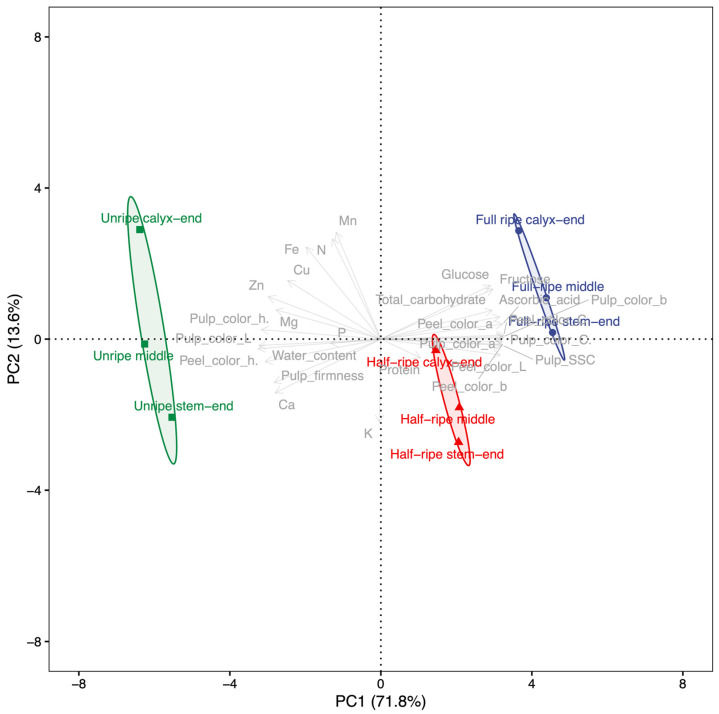
Principal component (PC) analysis scores of fruit qualities with PC scores of means on the first 2 PC axes for “Tainung No. 2” Red papaya (*Carica papaya*) at 3 parts across ripening stages with 95% confidence of ellipses for the mean of ripening stages. Percentages in parenthesis of each axis represent variances of each PC. Green—unripe at 16 weeks after anthesis (WAA); red—half-ripe at 18 WAA; blue—full-ripe at 20 WAA.

The fruit characteristics of papaya were analyzed by using principal component analysis (PCA) to identify patterns and relationships among variables. In this study, the PCA results showed that the fruit characteristics were distributed across each PC, with the PCs arranged according to the size of their variance, as shown in Figure 2. PC1 and PC2 accounted for the majority of the variance at 85.36%. Specifically, PC1 explained 71.78% of the variance and was associated with chromaticity, soluble solid contents, and ascorbic acid. These fruit characteristics had a high correlation value of approximately 0.98 on an absolute average, contributing to 59.78% of the variance in PC1. Notably, peel h°, pulp L, and pulp h° had negative correlations with PC1, while the other fruit characteristics had positive correlations. Overall, PC1 was indicative of papaya fruit ripening. In contrast, PC2 accounted for 13.58% of the variance and reflected the different parts of the fruit.

## 3. Discussion

In our result, the ratio of the length to the width of “Tainung No.2” was an average of 2.4 during the entire ripening stage, which is consistent with the general shape of hermaphroditic fruits [20]. In addition to this ratio, the characteristics associated with the fruit volume, including length and width, did not change during ripening. The growth of papaya fruit follows a sigmoidal pattern, with size development being completed before the onset of ripening [3]. This is due to the cessation of cell expansion and enlargement, with carbon sources being redirected toward metabolic changes such as the accumulation of pigments and soluble sugars [13,21]. This redistribution of carbon sources is critical for the development of desirable fruit qualities such as flavor, aroma, and color during ripening [13].

The degree of fruit ripeness is a consumer-driven trait and has significant importance during picking, packing, and transportation [18]. Fruit color is an essential indicator of ripeness in fresh fruits, including papaya [18] and mangoes [22], providing visual cues for marketable values. In this study, “Tainung No.2” papaya fruit showed distinct changes in both peel and pulp colors during ripening, with the colors evenly distributed throughout the fruit. Along with changes in color, the soluble solid contents increased while the firmness decreased during ripening, regardless of the fruit part. These changes are commonly observed during the ripening of various fruits, including papaya [18,23] and mangoes [22]. The softening of papaya fruit is highly correlated with the sweetening process that is associated with soluble solid contents, possibly due to the easier release of cellular contents in fully ripened tissue [23]. The results suggest that color changes and changes in soluble solid contents and firmness can be used as reliable indicators of papaya fruit ripeness.

The present study demonstrated that individual metabolites in papaya fruit exhibited unique accumulation patterns in different fruit parts during ripening. Although research on differences among tissue zones has been conducted on apples [13,19,24,25], there is limited information to determine the characteristics of each part of papaya fruit, with no comparative data in previous studies on papaya fruits. Doerflinger, Miller, Nock, and Watkins [13] compared the carbohydrate concentration of the stem-end, middle, and calyx-end in three apple cultivars (“Empire”, “Honeycrisp”, and “Gala”) during ripening. In the “Empire” cultivar, the concentration of starch was the highest at the calyx-end and the lowest at the stem-end. “Honeycrisp” and “Empire” had the highest concentration of sorbitol in the calyx-end, whereas the concentration was highest in the stem-end in “Gala”. The distribution differences of glucose, fructose, and sucrose were similar in all three cultivars: higher fructose and glucose concentrations in the stem-end and higher sucrose concentrations in the calyx-end of the fruit. In papaya fruit, soluble sugars are accumulated mainly when the fruit remains attached to the plant [23]. At the unripe stage, glucose is prevalent among the soluble sugars, and during ripening, sucrose becomes the predominant sugar with the modification of the soluble sugar profiles [26]. Chan Jr and Kwok [27] reported that sucrose levels varied from 1.8% to 8.0% during ripening. In this study, “Tainung No. 2” Red papaya fruit accumulated more total carbohydrates in the stem-end than in the other parts. The main soluble sugars in papaya are glucose, fructose, and glucose [28]; however, their compositions vary among cultivars [28]. In “Tainung No. 2” papaya fruit, only fructose and glucose were identified during ripening and were accumulated more in the calyx-end than in other parts. In addition to carbohydrates, more protein was accumulated at the stem-end. These differences could be associated with different development rates in different tissue zones. An increase in total primary amounts is associated with carbon allocation from the photosynthetic organs before ripening. Meanwhile, the accumulation of soluble sugars is one of the ripening processes. In papaya fruit, the accumulation rate rapidly increases during ripening [29]. Therefore, changes in primary metabolites are more pronounced and active during the ripening process of “Tainung No. 2” papaya fruit. The differences in the structure and size of cells could also lead to developmental differences within the fruit. However, such differences vary by each species [13] and its cultivars [30,31]. 

Establishing nutrient absorption and accumulation patterns is critical for planning optimum nutrient supply and improving the influence on fruit quality [32]. Macro- and micro-element accumulation shows dynamic variance during ripening. In this study, all elements except N at the stem-end decreased or were maintained at the full-ripe stage, despite different patterns at the half-ripe stage among fruit parts. The decreases in the elements have also been shown in various fruits, including papaya [12] and apple fruit [33,34]. In apple pulp, when fruits are compared approximately 60 and 120 days after full bloom for the elements N, P, K, Mg, and S, there is a slight decrease, but Ca, Fe, Cu, Mn, and B contents had no significant differences [34]. In papaya pulp, Ca, K, P, and Mg decreased to 75.56%, 38.67%, 66.46%, and 50.00%, respectively, at the full-ripe stage, compared to the unripe stage. [12]. 

All papaya fruit samples were distinctly divided according to fruit characteristics. PCA revealed that the ripening stage was accounted for by PC1, while different fruit parts were accounted for by PC2. PC1 was associated with chromaticity, soluble solid contents, and ascorbic acid, making it a suitable indicator for selecting the most appropriate stage of ripening based on these characteristics. However, it is crucial to consider both fruit characteristics and fruit parts when evaluating the quality and nutritional value of papaya fruit. These findings suggest that the ripening process and nutrient accumulation in papaya fruit are complex and occur differently in different parts of the fruit, highlighting the importance of considering both fruit characteristics and fruit parts when evaluating the quality and nutritional value of papaya fruit.

## 4. Materials and Methods

### 4.1. Plant Materials

“Tainung No. 2” papaya seeds were collected from the Research Institute of Climate Change and Agriculture, the National Institute of Horticultural and Herbal Science, Jeju, Republic of Korea (33° 28 N′, 126° 31′ E). The seeds were sown on 10 November 2015 in black plastic pots (200 mm in diameter, 300 mm in length, and 30 L in volume) in a medium containing 90% commercial grow media (4% zeolite, 7% perlite, 6% vermiculite, 68% coco peat, and 14% peat moss) (Baroker, Seoul Bio Co., Ltd., Gyeongju, Republic of Korea) and 10% coarse sand (by volume), according to Joa et al. [35]. Plantlets were grown in a greenhouse of an experimental orchard in the Institute of Climate Change and Agriculture and then transplanted on 8 June 2016 into the ground of the same greenhouse. These trees were cultivated according to the standard guidelines for papaya cultivation [35]: the minimum temperature during winter was maintained at 15 °C in the greenhouse by using hot air blowers to prevent papaya trees from chilling injury.

The fruit was harvested in July 2018, owing to a short juvenile phase [5], and was categorized into 3 ripening stages based on weeks after anthesis (WAA), according to Zuhair, Aminah, Sahilah, and Eqbal [11]: (1) unripe at 16 WAA, (2) half-ripe at 18 WAA, and (3) full-ripe at 20 WAA. At each stage, a total of nine fruits were harvested in three biological replicates. After the length (mm), width (mm), and weight (g) of each fruit were measured, each fruit was dissected into three parts of equal lengths: stem-end, middle, and calyx-end for further analyses.

### 4.2. Determination of Colors, Soluble Solid Contents, and Firmness

The peel and pulp colors of papaya fruits were measured at the stem-end, middle, and calyx-end at the three ripening stages by using a spectrophotometer (CM700d, Minolta Co., Osaka, Japan) and described by the CIE L*, a*, and b* color space coordinates [36]. The L* value represents the lightness of colors, ranging from 0 to 100 (0, black; 100, white). The a* value was negative for green and positive for red. The b* value was negative for blue and positive for yellow. The values were measured at three midpoint regions of each fruit part. In addition to the CIE coordinates, the hue angle (h°) was calculated as tan−1 (b*/a*), which implies visual color appearance; 0° was represented by red-purple; 90° was represented by yellow; 180° was represented by bluish-green; 270° was represented by blue. The chroma (C*) was computed as (a*^2^ + b*^2^) × 1/2, which describes the quality of the color intensity or saturation. 

The soluble solid contents (°Brix) were measured by using a digital refractometer (HI98801, Hanna Instruments Inc., Woonsocket, RI, USA). Peel firmness was measured by using a texture analyzer (TA-XT express, Stable Micro System, Godalming, UK). A puncture test was conducted by using a 2.0 mm diameter cylindrical probe (Stable Micro System) at 3 different points along the fruit equator with a cross-head speed of 2.0 mm s^−1^ and a penetration depth of 5.0 mm.

### 4.3. Determination of Water Proportion and Carbohydrate, Protein, and Lipid Contents 

The fruit was dehydrated at 105 °C in a drying oven up to a constant weight to determine the proportion of water content compared with the total fresh weight of each sample, which was expressed as a percentage (%). To determine carbohydrate, protein, and lipid contents, all the samples were lyophilized by using a freeze dryer and finely ground by using a mortar and pestle. Total carbohydrate content was determined according to the procedure of Jermyn [37]. Protein concentration was determined by using Micro-Kjeldahl’s apparatus [38]. Lipid concentration was determined by using the Soxhlet extraction method [39]. All analyses were performed according to the official analysis methods of the Association of Official Analytical Chemists (AOAC) [40].

### 4.4. Determination of Free Sugars

Free sugars were extracted according to the method described by Oh et al. [41] with some modifications. In total, 3 grams of ground fruits was added to 50 mL of 50% acetonitrile. The samples were extracted thrice via the ultrasonic extraction method for 6 h. The extract was diluted and filtered through Sep-Pak C18 cartridge column (Waters, Milford, MA, USA) and a 0.45 μm pore size micro-filter (Woongki Science Co., Ltd., Seoul, Republic of Korea). After the sample preparation, the free sugars were separated in a Prevail TM Carbohydrate ES column (4.6 × 250 mm, 5 μm, Grace, Deerfield, IL, USA) that was equipped with an HPLC system (Waters 2695, Waters Associate Inc., Milford, MA, USA). The eluents were passed through the column at a flow rate of 0.8 mL/min by using acetonitrile: distilled water (70:30, *v*/*v*). The chromatographic peak corresponding to free sugar was identified by comparing the retention times with those of glucose, fructose, and sucrose (Sigma, St. Louis, MO, USA) as standards. Concentrations were calculated by using the calibration curve that was generated from the standard solutions.

### 4.5. Determination of Ascorbic Acids

Ascorbic acid was extracted by using the procedure described by Rizzolo et al. [42], with some modifications. Thirty grams of samples were added to 25 mL of 6% metaphosphoric acid. The sample was homogenized and then centrifuged at 10,000× *g* at 4 °C for 10 min. The supernatant was filtrated through filter paper (Whatman No. 4, Whatman plc, Kent, UK), a membrane filter (MF-milliporeTM 0.22 μm, Merck KGaA, Darmstadt, Germany), and a Waters Sep-Pak C18 column (Waters, Milford, CT, USA). Ascorbic acids in the prepared samples were separated by using a Waters Bonda Pak NH2 (3.9 × 300 mm) column (Waters) that was equipped with an HPLC system (Waters TM 600 controller, Waters TM 616 Pump, Waters 717 plus auto Sampler, Waters TM 486 tunable absorbance detector). The eluent was passed through the column by using 5 mM KH_2_PO_4_ (pH 4.6): acetonitrile (30:70, *v*/*v*) (A) and acetonitrile (B) by using a linear gradient from 100% A to 40% A in 50 min at a flow rate of 1 mL/min. The chromatographic peak corresponding to ascorbic acid in the samples was identified by comparing its retention times with those of the standard (Sigma). Concentrations were calculated by using the calibration curve that was generated from standard solutions prepared for the standard. The limit of detection (LOD) and limit of quantification (LOQ) for ascorbic acid was determined to ascertain the linearity of the method, as previously described by [43,44]; LOD of 0.003 μg∙μL^−1^ and LOQ of 0.013 μg∙μL^−1^.

### 4.6. Determination of Macro- and Micro-elements 

The proportions of nitrogen and phosphorus contents were determined by using the Kjeldahl method [45] and the molybdenum blue method [46], respectively. In total, 3 grams of fruit was weighed and heated in a furnace for 4 h at 550 °C. The crucible was cooled down in a desiccator and dissolved in 2.5 mL of a decomposition solution (HNO_3_:H_2_SO_4_:HClO_4_ (10:1:4, *v*/*v*/*v*)). The solution was filtered and diluted up to 100 mL by using distilled water. Exchangeable cations (K, Ca, and Mg) and minerals (Fe, Mn, Zn, and Cu) were determined by inductively coupled plasma spectrophotometry (ICP-Integra XL, GBC Scientific Equipment Pty Ltd., Braeside, Australia) following the procedure of Zarcinas et al. [47]. The results were obtained by using a working standard of 1000 ppm for each sample [41].

### 4.7. Statistical Analyses

To determine statistical significance, data were analyzed by using the R 4.2.2 [48]. Statistical significances were determined by using a two-way analysis of variance with the agricolae v1.3-5 package [49]. The means of fruit characteristics were compared by using Duncan’s multiple range tests at *p* < 0.05.

PCA was performed on fruit characteristics by using the mean values of replicate samples. The Pearson correlation coefficient was used to calculate the correlation matrix among the fruit characteristics, with a significance level of *p* < 0.05. The PCA was conducted with raw data by using the factoextra v1.0.7 package in R [50]. The variables were standardized by subtracting the mean and then dividing the result by the standard deviation of each original variable to assign each weight in the analysis, according to Abdi and Williams [51].

## 5. Conclusions

Changes in color, soluble solid contents, and firmness can be reliable indicators of papaya fruit ripeness. Moreover, individual metabolites showed unique accumulation patterns in different fruit parts during ripening, with the stem-end accumulating more total carbohydrates and protein. The nutrient accumulation patterns exhibited dynamic variance during ripening, emphasizing the need to consider both fruit characteristics and parts when evaluating the quality and nutritional value of papaya fruit. Overall, our findings provide valuable insights into the ripening process and nutrient accumulation in papaya fruit, which can help optimize nutrient supply and improving fruit quality.

## Figures and Tables

**Table 1:** Fruit length (mm), width (mm), the ratio of the length to the width, and weight (g FW) of “Tainung No. 2” Red papaya (*Carica papaya*) at 3 parts across ripening stages: unripe at 16 weeks after anthesis (WAA), half-ripe at 18 WAA, and full-ripe at 20 WAA.

Stage	Length	Width	Ratio of Length to Width	Weight
	mm			g
Unripe	25.9 ± 1.40 ^a†^	9.6 ± 0.54 ^a^	2.7 ± 0.27 ^a^	865.9 ± 123.92 ^a^
Half-ripe	25.8 ± 0.80 ^a^	10.3 ± 1.11 ^a^	2.5 ± 0.32 ^a^	997.9 ± 110.92 ^a^
Full-ripe	19.2 ± 5.08 ^a^	10.0 ± 0.73 ^a^	1.9 ± 0.67 ^a^	699.6 ± 200.26 ^a^

^†^ Different letters within columns indicate significant differences by Duncan’s multiple range test at *p* < 0.05.

**Table 2:** Peel chromaticity of “Tainung No. 2” Red papaya (*Carica papaya*) at 3 parts across ripening stages: unripe at 16 weeks after anthesis (WAA), half-ripe at 18 WAA, and full-ripe at 20 WAA.

Stage	Position	Peel Chromaticity				
		L*	a*	b*	h°	C*
Unripe	Stem-end	35.5 ± 1.66 ^b†^	−8.1 ± 0.45 ^cd^	9.3 ± 0.90 ^b^	131.2 ± 1.60 ^a^	12.4 ± 0.95 ^b^
	Middle	34.3 ± 0.58 ^b^	−8.7 ± 0.43 ^cd^	9.0 ± 0.20 ^b^	132.2 ± 1.13 ^a^	12.2 ± 0.41 ^b^
	Calyx-end	34.9 ± 1.01 ^b^	−8.8 ± 1.35 ^d^	12.2 ± 3.36 ^b^	126.4 ± 3.71 ^a^	15.0 ± 3.48 ^b^
Half-ripe	Stem-end	58.0 ± 4.55 ^a^	18.2 ± 12.01 ^ab^	51.0 ± 6.20 ^a^	71.4 ± 1.12 ^bc^	54.8 ± 8.55 ^a^
	Middle	55.6 ± 2.21 ^a^	13.1 ± 14.08 ^abc^	48.5 ± 2.16 ^a^	75.9 ± 11.60 ^bc^	51.3 ± 6.48 ^a^
	Calyx-end	55.7 ± 2.61 ^a^	10.1 ± 12.46 ^bcd^	47.6 ± 3.48 ^a^	79.1 ± 13.85 ^b^	49.5 ± 6.50 ^a^
Full-ripe	Stem-end	56.8 ± 1.85 ^a^	32.5 ± 2.25 ^a^	53.9 ± 3.29 ^a^	58.9 ± 12.64 ^bc^	62.9 ± 3.74 ^a^
	Middle	54.5 ± 1.96 ^a^	31.7 ± 1.42 ^a^	51.5 ± 2.62 ^a^	58.4 ± 1.60 ^bc^	60.5 ± 2.44 ^a^
	Calyx-end	56.0 ± 1.81 ^a^	33.5 ± 0.95 ^a^	51.0 ± 3.79 ^a^	56.7 ± 1.24 ^c^	61.1 ± 3.68 ^a^

^†^ Different letters within columns indicate significant differences by Duncan’s multiple range test at *p* < 0.05. L*—the lightness of colors, ranging from 0 to 100 (0, black; 100, white); a*—negative for green and positive for red; b*—negative for blue and positive for yellow; h°—hue angle calculated as tan^−1^ (b*/a*); C*—chroma calculated as (a*^2^ + b*^2^) × 1/2.

**Table 3:** Pulp chromaticity, soluble solid contents, and firmness (N) of “Tainung No. 2” Red papaya (*Carica papaya*) at 3 parts across ripening stages unripe at 16 weeks after anthesis (WAA), half-ripe at 18 WAA, and full-ripe at 20 WAA.

Stage	Position	Chromaticity					SSC	Firmness
		L*	a*	b*	h°	C*	°Brix	N
Unripe	Stem-end	75.1 ± 1.12 ^a†^	−3.7 ± 0.98 ^b^	23.7 ± 3.40 ^c^	98.8 ± 1.10 ^a^	24.0 ± 3.51 ^c^	4.6 ± 0.31 ^b^	11.1 ± 0.65 ^a^
	Middle	74.4 ± 3.10 ^a^	−4.2 ± 0.74 ^b^	24.3 ± 3.16 ^c^	99.8 ± 1.13 ^a^	24.7 ± 3.21 ^c^	3.4 ± 0.92 ^b^	11.7 ± 0.91 ^a^
	Calyx-end	75.7 ± 3.01 ^a^	−3.3 ± 0.87 ^b^	22.2 ± 3.35 ^c^	98.5 ± 0.92 ^a^	22.4 ± 3.44 ^c^	3.7 ± 0.21 ^b^	11.5 ± 0.71 ^a^
Half-ripe	Stem-end	57.8 ± 0.46 ^b^	28.6 ± 1.23 ^a^	38.5 ± 1.12 ^b^	53.4 ± 1.98 ^b^	48.0 ± 0.20 ^ab^	11.3 ± 0.46 ^a^	8.2 ± 1.86 ^a^
	Middle	57.8 ± 1.14 ^b^	30.1 ± 2.81 ^a^	40.2 ± 3.77 ^ab^	53.2 ± 2.17 ^b^	50.3 ± 4.32 ^ab^	9.7 ± 0.50 ^a^	7.5 ± 4.14 ^a^
	Calyx-end	58.5 ± 2.51 ^b^	28.6 ± 2.17 ^a^	38.1 ± 0.26 ^b^	53.1 ± 1.89 ^b^	47.6 ± 1.51 ^b^	9.4 ± 0.93 ^a^	8.5 ± 2.41 ^a^
Full-ripe	Stem-end	54.1 ± 4.10 ^b^	30.6 ± 4.04 ^a^	42.6 ± 2.45 ^ab^	54.5 ± 2.04 ^b^	52.5 ± 4.34 ^ab^	11.3 ± 1.80 ^a^	0.4 ± 0.29 ^b^
	Middle	55.2 ± 1.37 ^b^	31.9 ± 2.67 ^a^	46.7 ± 2.64 ^a^	55.7 ± 2.99 ^b^	56.6 ± 2.28 ^a^	11.7 ± 0.40 ^a^	0.3 ± 0.11 ^b^
	Calyx-end	51.3 ± 4.25 ^b^	33.7 ± 1.59 ^a^	42.3 ± 3.22 ^ab^	51.4 ± 2.44 ^b^	54.1 ± 2.77 ^ab^	11.4 ± 0.29 ^a^	0.4 ± 0.09 ^b^

^†^ Different letters within columns indicate significant differences by Duncan’s multiple range test at *p* < 0.05. L*—the lightness of colors, ranging from 0 to 100 (0, black; 100, white); a*—negative for green and positive for red; b*—negative for blue and positive for yellow; h°—hue angle calculated as tan^−1^ (b*/a*); C*—chroma calculated as (a*^2^ + b*^2^) × 1/2. SSC—soluble solid contents.

**Table 4:** Water, carbohydrate, protein, and lipid contents of “Tainung No. 2” Red papaya (*Carica papaya*) at 3 parts across ripening stages: unripe at 16 weeks after anthesis (WAA), half-ripe at 18 WAA, and full-ripe at 20 WAA.

Stage	Position	Water	Carbohydrate	Protein	Lipid
		%		g/100 g FW	
Unripe	Stem-end	94.0 ± 0.03 ^a†^	4.5 ± 0.02 ^e^	1.06 ± 0.03 ^cd^	nd
	Middle	93.7 ± 0.15 ^a^	5.1 ± 0.15 ^d^	0.91 ± 0.01 ^de^	nd
	Calyx-end	93.9 ± 0.02 ^a^	4.5 ± 0.05 ^e^	1.15 ± 0.05 ^bc^	nd
Half-ripe	Stem-end	92.6 ± 0.02 ^b^	5.4 ± 0.05 ^d^	1.29 ± 0.01 ^ab^	nd
	Middle	92.5 ± 0.04 ^b^	6.1 ± 0.02 ^c^	0.93 ± 0.03 ^de^	nd
	Calyx-end	92.3 ± 0.03 ^b^	5.9 ± 0.03 ^c^	1.11 ± 0.01 ^c^	nd
Full-ripe	Stem-end	90.6 ± 0.08 ^d^	7.4 ± 0.08 ^a^	1.39 ± 0.02 ^a^	nd
	Middle	91.4 ± 0.04 ^c^	6.7 ± 0.03 ^b^	1.20 ± 0.02 ^bc^	nd
	Calyx-end	91.6 ± 0.05 ^c^	6.9 ± 0.04 ^b^	0.88 ± 0.01 ^e^	nd

^†^ Different letters within columns indicate significant differences by Duncan’s multiple range test at *p* < 0.05. Nd—not detected.

**Table 5:** Changes in the content of soluble sugars of “Tainung No. 2” Red papaya (*Carica papaya*) pulp at 3 parts across ripening stages: unripe at 16 weeks after anthesis (WAA), half-ripe at 18 WAA, and full-ripe at 20 WAA.

Stage	Position	Fructose	Glucose	Sucrose
			g∙100 g^−1^ FW	
Unripe	Stem-end	1.1 ± 0.01 ^e†^	1.3 ± 0.00 ^d^	nd
	Middle	1.1 ± 0.08 ^e^	1.3 ± 0.03 ^d^	nd
	Calyx-end	1.4 ± 0.07 ^e^	1.6 ± 0.03 ^d^	nd
Half-ripe	Stem-end	1.8 ± 0.17 ^d^	2.0 ± 0.07 ^c^	nd
	Middle	1.9 ± 0.23 ^d^	2.1 ± 0.09 ^c^	nd
	Calyx-end	2.1 ± 0.12 ^c^	2.4 ± 0.04 ^c^	nd
Full-ripe	Stem-end	2.5 ± 0.01 ^bc^	2.9 ± 0.01 ^b^	nd
	Middle	2.7 ± 0.14 ^ab^	3.1 ± 0.17 ^ab^	nd
	Calyx-end	2.9 ± 0.05 ^a^	3.4 ± 0.05 ^a^	nd

^†^ Different letters within columns indicate significant differences by Duncan’s multiple range test. Nd—not detected.

**Table 6:** Changes in the content of ascorbic acid at each part of “Tainung No. 2” Red papaya (*Carica papaya*) pulp at 3 parts across ripening stages: unripe at 16 weeks after anthesis (WAA), half-ripe at 18 WAA, and full-ripe at 20 WAA.

Stage	Position	Ascorbic acid
		mg∙100 g^−1^ FW
Unripe	Stem-end	8.7 ± 0.43 ^c†^
	Middle	6.8 ± 0.20 ^c^
	Calyx-end	6.9 ± 0.37 ^c^
Half-ripe	Stem-end	21.9 ± 0.23 ^b^
	Middle	23.2 ± 0.53 ^b^
	Calyx-end	22.1 ± 1.10 ^b^
Full-ripe	Stem-end	32.7 ± 0.97 ^a^
	Middle	31.4 ± 0.97 ^a^
	Calyx-end	30.7 ± 1.13 ^a^

^†^ Different letters within columns indicate significant differences by Duncan’s multiple range test.

**Table 7:** Changes in macronutrients of “Tainung No. 2” Red papaya (*Carica papaya*) pulp at 3 parts across ripening stages: unripe at 16 weeks after anthesis (WAA), half-ripe at 18 WAA, and full-ripe at 20 WAA.

Stage	Position	N	P	K	Ca	Mg
		mg∙g^−1^ FW				
Unripe	Stem-end	0.7 ± 0.01 ^def†^	0.5 ± 0.02 ^a^	3.3 ± 0.13 ^a^	0.4 ± 0.03 ^a^	0.15 ± 0.010 ^b^
	Middle	0.8 ± 0.01 ^bcd^	0.4 ± 0.02 ^bc^	2.8 ± 0.10 ^b^	0.4 ± 0.04 ^a^	0.20 ± 0.010 ^a^
	Calyx-end	1.2 ± 0.07 ^a^	0.4 ± 0.03 ^b^	2.6 ± 0.13 ^b^	0.3 ± 0.02 ^b^	0.16 ± 0.010 ^b^
Half-ripe	Stem-end	0.7 ± 0.08 ^ef^	0.4 ± 0.01 ^ab^	3.5 ± 0.19 ^a^	0.3 ± 0.02 ^bc^	0.09 ± 0.000 ^e^
	Middle	0.6 ± 0.02 ^f^	0.3 ± 0.01 ^c^	2.7 ± 0.14 ^b^	0.3 ± 0.02 ^bc^	0.12 ± 0.000 ^cd^
	Calyx-end	0.8 ± 0.04 ^cde^	0.4 ± 0.02 ^b^	2.6 ± 0.10 ^b^	0.2 ± 0.01 ^cd^	0.13 ± 0.000 ^c^
Full-ripe	Stem-end	0.9 ± 0.07 ^bc^	0.4 ± 0.04 ^bc^	3.3 ± 0.25 ^a^	0.2 ± 0.01 ^d^	0.09 ± 0.000 ^e^
	Middle	0.7 ± 0.01 ^def^	0.3 ± 0.03 ^c^	2.5 ± 0.19 ^b^	0.1 ± 0.00 ^d^	0.11 ± 0.000 ^de^
	Calyx-end	0.9 ± 0.07 ^b^	0.4 ± 0.02 ^ab^	2.5 ± 0.14 ^b^	0.1 ± 0.00 ^d^	0.13 ± 0.000 ^c^

^†^ Different letters within columns indicate significant differences by Duncan’s multiple range test.

**Table 8:** Changes in micronutrients of “Tainung No. 2” Red papaya (*Carica papaya*) pulp at 3 parts across ripening stages (unripe, half-ripe, and full-ripe).

Stage	Position	Cu	Fe	Mn	Zn
		μg∙g^−1^ FW			
Unripe	Stem-end	0.28 ± 0.033 ^b†^	2.68 ± 0.074 ^bcd^	0.09 ± 0.005 ^cd^	1.36 ± 0.095 ^b^
	Middle	0.30 ±0.027 ^b^	2.94 ± 0.134 ^ab^	0.10 ± 0.010 ^cd^	1.78 ± 0.164 ^a^
	Calyx-end	0.42 ± 0.029 ^a^	3.12 ± 0.159 ^a^	0.27 ± 0.018 ^a^	1.73 ± 0.154 ^a^
Half-ripe	Stem-end	0.23 ± 0.024 ^b^	2.19 ± 0.070 ^ef^	0.05 ± 0.004 ^d^	0.80 ± 0.042 ^c^
	Middle	0.22 ± 0.028 ^b^	2.14 ± 0.063 ^f^	0.05 ± 0.003 ^d^	0.77 ± 0.063 ^c^
	Calyx-end	0.30 ± 0.042 ^b^	2.41 ± 0.083 ^def^	0.07 ± 0.006 ^cd^	0.84 ± 0.068 ^c^
Full-ripe	Stem-end	0.24 ± 0.023 ^b^	2.51 ± 0.149 ^cde^	0.09 ± 0.016 ^cd^	0.81 ± 0.070 ^c^
	Middle	0.24 ± 0.010 ^b^	2.62 ± 0.060 ^bcd^	0.11 ± 0.019 ^c^	0.86 ± 0.038 ^c^
	Calyx-end	0.24 ± 0.010 ^b^	2.83 ± 0.075 ^abc^	0.19 ± 0.040 ^b^	1.06 ± 0.085 ^c^

^†^ Different letters within columns indicate significant differences by Duncan’s multiple range test.

## Data Availability

The data presented in the study are included in this article. Further inquiries can be directed to the corresponding author.
